# An Overview of Histamine and Other Biogenic Amines in Fish and Fish Products

**DOI:** 10.3390/foods9121795

**Published:** 2020-12-03

**Authors:** Pierina Visciano, Maria Schirone, Antonello Paparella

**Affiliations:** Faculty of Bioscience and Technology for Food, Agriculture and Environment, University of Teramo, Via R. Balzarini, 1, 64100 Teramo, Italy; pvisciano@unite.it (P.V.); apaparella@unite.it (A.P.)

**Keywords:** biogenic amines, histamine food poisoning, outbreaks, seafood safety

## Abstract

The occurrence of biogenic amines in fish is directly associated with microorganisms with decarboxylase activity. These compounds are generally detoxified by oxidases in the intestinal tract of humans, but some conditions, such as alcohol consumption, enzyme deficiency, or monoamino-oxidase antidepressant use, can make their intake by food dangerous. Due to its toxicity, histamine is the unique biogenic amine with regulatory limits for fishery products. This review focuses on biogenic amines in fish, with a detailed picture of the number of alert notifications or intoxication events reported in the last years. The favoring conditions for their formation, as well as the main preventive and control measures to ensure public health, are also reviewed.

## 1. Introduction

Biogenic amines (BAs) are nitrogenous compounds resulting from the free amino acid decarboxylation or the amination of carbonyl-containing organic compounds through the metabolism of different microorganisms. Thus, their accumulation in food can be considered a good indicator of spoilage [1]. BAs are distinguished based on chemical structure into heterocyclic, aliphatic, or aromatic compounds (Table 1), or based on the number of amine groups into monoamines (tyramine and phenylethylamine), diamines (histamine, putrescine, and cadaverine), or polyamines (spermidine and spermine) [2].

Fish products constitute an important part of the human diet because they are an excellent source of nutrients, including proteins, vitamins, salt minerals, and polyunsaturated fatty acids [3]. Nevertheless, they are very perishable due to postmortem modifications followed by the formation of spoilage compounds, such as organic acids, aldehydes and ketones, alcohols, sulfides, and BAs [4]. The inappropriate storage of fish and/or temperature abuse can lead to BA formation due to microbial enzymatic activities. 

Gram-positive and -negative bacteria associated with fish spoilage can produce BAs. They are generally located on skin, gills, or in the gastrointestinal tract [5,6,7,8,9,10,11] (Table 2), and can spread to muscle tissue during butchering or gutting through rupture or spillage of gastric contents [12]. The most frequent species belong to *Enterobacteriaceae* and include mesophilic and psychrotolerant bacteria, such as *Morganella*, *Enterobacter*, *Hafnia*, *Proteus,* and *Photobacterium* [13]. Also, *Pseudomonas* spp. and lactic acid bacteria belonging to *Lactobacillus* and *Enterococcus* genera can cause BA formation [14]. 

The amounts of BAs ingested by food are regulated in the human organism by a detoxification system formed mainly by mono and diamino oxidases. High intake of BAs or the presence of factors such as the use of alcohol or medication that reduce the effectiveness of such detoxifying enzymes, rather than a genetic deficiency, can lead to intoxication with different symptoms depending on the type of BA. Histamine is the most toxic, as it can act as neurotransmitter and vasodilator, causing headache, hypotension, heart palpitations, asthma attacks, and cutaneous (edema and flushing of the face, neck, and upper arms) or gastrointestinal (difficulties in swallowing, vomiting, and diarrhea) effects [7]. Bronchospasm, respiratory distress, and vasodilatory shock are also described [15]. A detailed summary of histamine intoxication is reported in Table 3. Instead, tyramine may increase the cardiac frequency or cause respiratory disorders, but also nausea and vomiting, while phenylethylamine can be a migraine inductor. Further symptoms associated with tyramine and phenylethylamine are hypertension and cerebral hemorrhage. Even if putrescine and cadaverine are not toxic, they can potentiate the adverse effects of the above cited BAs, as they favor their adsorption or interfere with the detoxification system [16,17]. 

In the literature, several reviews are reported on BAs in fish. Some authors [2,6,18] described in detail the content of histidine in some fish species, the decarboxylation reactions, some indexes of quality, and toxicological effects of many BAs, not only histamine, alongside the presence of these compounds in various foods. Instead, the focus of this review is the statement of outbreaks reported in the Member States of European Union (EU) and the notifications of the Rapid Alert System of Food and Feed (RASFF). In addition, factors affecting BA formation are also discussed.

## 2. Biogenic Amines in Seafood: Focus on Scombroid Poisoning Outbreaks

Histamine, tyramine, putrescine, and cadaverine are the most common BAs found in fish and derive from decarboxylation of corresponding free amino acids by microorganisms [18]. Their accumulation depends on the presence of the precursor amino acids, the growth or activity of decarboxylating bacteria, and a favorable environment. The main factors that influence the microbiota and its enzymatic activity are temperature, pH, water activity, oxygen availability, concentration of NaCl, some additives, and competition among microorganisms. The combination of these factors can be responsible of the variability of BA content within the same batch, and also within individual fish [12,19]. 

In the flesh of fish, BAs can be considered an indicator of good handling and storage procedures, and some authors reported different chemical indices for fish quality as combinations of amines [20]. However, the levels of different BAs are associated with the predominant muscle type in fish. Fish with dark muscles have more histidine content compared to those with white muscles, and therefore they accumulate more histamine when kept under elevated temperatures. On the contrary, fish belonging to the second category (white muscles) can show high cadaverine and putrescine concentrations due to poor handling, but also temperature abuse. For cephalopods, agmatine is used as a quality indicator, while in the case of crustaceans, shrimp, and lobsters, putrescine and cadaverine are considered [21]. Individual BA or a combination of various amines are considered as a quality index of fish freshness. Some authors reported that a histamine content lower than a value of 10 mg/kg denotes fish of good quality, while concentrations between 30 and 50 mg/kg represent important and definite deterioration, respectively [22]. Instead, the consumption of meals with histamine concentrations of 8–40 mg can cause only slight intoxication, while values of 40–100 mg or higher than 100 mg are associated with intermediate and severe intoxication, respectively [2]. The Food and Drug Administration (FDA) established a defect action level of 50 mg/kg for histamine (according to the revised Guidance in year 2020) [23], whereas the Commission Regulation (EC) No 2073/2005, which is currently in force, fixed maximum levels of 200 and 400 mg/kg for raw fish and fishery products subjected to enzyme maturation treatment in brine, respectively. These limits apply to the fish families *Scombridae*, *Scomberosocidae, Engraulidae*, *Clupeidae*, *Coriphaenidae,* and *Pomatomidae*, with high concentrations of free histidine in the muscle tissue. Some outbreak reports [24] and scientific studies confirmed that fish with low levels of the precursor amino acid showed no toxic histamine content in their flesh, whereas the opposite situation was described by Visciano et al. [25] in fish experimentally subjected to temperature abuse and belonging to the above cited families with regulatory limits.

Scombroid poisoning occurs worldwide and the largest numbers of such events are described in the United States, the United Kingdom, Australia, and Japan. Up to 40% of foodborne outbreaks reported in Europe and the United States can be ascribed to histamine intoxication [26]. In Table 4, the histamine poisoning and human cases in the EU from 2010 to 2018 are shown. Even if a positive trend was observed during the years 2010–2017, a sudden decrease was described in 2018, probably due to the lack of case reports from some Member States [27,28,29,30,31,32,33,34,35].

**Figure 1:**
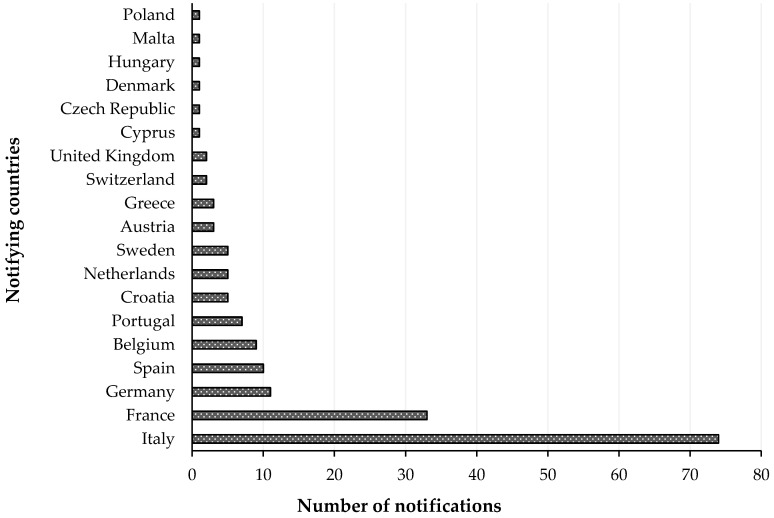
List of notifying countries and number of notifications for histamine presence in “fish and fish products” by the Rapid Alert System for Food and Feed from 1 January 2015 to 31 August 2020.

**Figure 2:**
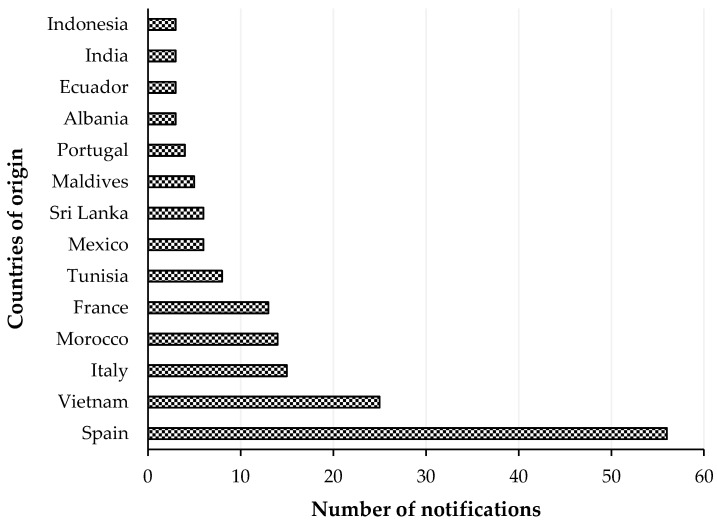
List of countries of origin with notifications of histamine presence in “fish and fish products” by the Rapid Alert System for Food and Feed from 1 January 2015 to 31 August 2020.

**Figure 3:**
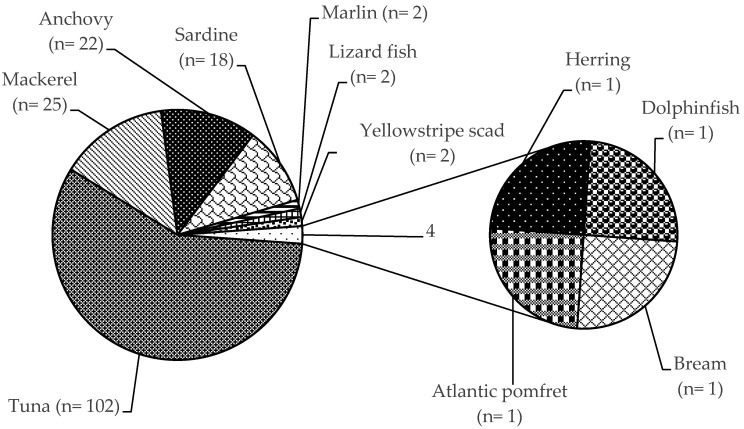
Distribution of histamine in fish species according to the Rapid Alert System for Food and Feed from 1 January 2015 to 31 August 2020.

**Figure 4:**
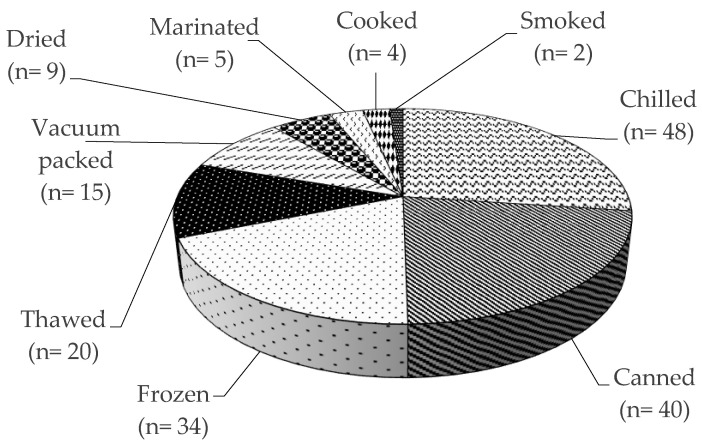
Presence of histamine in different fish products according to the Rapid Alert System for Food and Feed from 1 January 2015 to 31 August 2020.

RASFF organized among the EU Member States for the notification of risks to human health was established by the Regulation (EC) No 178/2002, with histamine representing one of the most common causes of such notifications. The list of notifying countries and the countries of origin of notifications for histamine presence in fish products from 2015 to 2020 are shown in Figure 1 and Figure 2, respectively. Italy is the major notifying Member State, with a total of 74 alert or information for attention notifications, followed by France, with 33. Instead, the main origin of fish and fish products contaminated by histamine is linked to Spain (56 cases). Most notifications derived from the analysis of products showed high concentrations of histamine (Table 4), whereas some cases (n = 22) were associated with foodborne outbreaks generally occurring in the notifying country. The highest percentage of both alert (30.2%) and information for attention (34.4%) notifications reported a histamine content (Table 5) that slightly exceeded the maximum limits set by the Commission Regulation (EC) No 2073/2005. Instead, the highest values, i.e., >1000 or even >2000 mg/kg, referred to scombroid poisoning caused by chilled or canned fish, particularly regarding tuna species. In Figure 3 and Figure 4, the fish species and types of fish products (i.e., raw or processed) involved in the RASFF reports from 2015 to August 2020 are shown. Tuna is the most representative species because it presents high concentrations of histidine in muscle tissue for its high speed and long duration swimming as predator fish [36]. Moreover, harvesting practices, such as longlining and gillnetting, can contribute to histamine formation due to the long period in which the fish remains in the sea before it is brought onboard the vessel. Such conditions are particularly dangerous for tuna species, which can generate heat into their body that exceeds the environmental temperature, thereby favoring the growth of histamine-forming microorganisms [23].

## 3. Factors Affecting Biogenic Amine Formation in Fresh and Processed Seafood 

Several parameters associated with food (i.e., temperature, NaCl, redox potential, pH, water activity (a_w_), oxygen supply, etc.), as well as the hygienic conditions of manufacturing practices, can play a significant role in BA formation. The highest production by microorganisms occurs at temperatures ranging from 20 to 37 °C, so that the cold chain maintenance after harvesting of fish may prevent BA accumulation by reducing both bacterial growth and enzyme activity [37]. However, some decarboxylases continue their functions even if the microbial cells are not active. This phenomenon was demonstrated for histidine decarboxylase in Gram-negative bacteria such as *Morganella morganii*, *Photobacterium damselae*, *Photobacterium phosphoreum,* and *Raoultella planticola* [38]. Moreover, the specific metabolism of the microorganisms, the variability of strains belonging to the same species, as well as the complex matrix of analysis can influence decarboxylase responses to the environmental factors [39]. 

Even if it is important to ice fish as quickly as possible after catching, this practice cannot prevent/inhibit enzyme activities or microbial spoilage. Superchilling is a low temperature-based technique that consists in the decrease of temperature to 1–2 °C below freezing point (i.e., 0 °C) so that only a minor part of the water content of fish is frozen [40]. It reduces most autolytic and microbial reactions compared with normal chilling, and therefore its application can extend the shelf-life of many fish products [41]. Also, freezing at temperatures ranging from −18 to −30 °C inhibits microbial growth, but some enzymatic and nonenzymatic reactions can persist at lower rates and the formation of large ice crystals during such process may increase the risk of texture damage, loss of water holding capacity, and oxidation [42]. 

Besides temperature, pH and redox potential of the medium can influence amino acid decarboxylase activity. At low pH, microorganisms are more induced to generate decarboxylases as a protective mechanism from acidity, whereas conditions bringing about a diminished redox potential, enhance histamine formation [37]. *Photobacterium* spp., enterobacteria, and pseudomonads produce low quantities of BAs when NaCl concentrations correspond to 4–5%, even if the decarboxylation reactions are still operating [39]. 

Some other hurdles can be useful to preserve the characteristics and shelf-life of fish products, i.e., application of osmotic dehydration process, preservatives, and competitive microorganisms, such as lactic acid bacteria (LAB). The use of LAB and their metabolites as biopreservation techniques received much attention over the last two decades [43]. They are generally used for their ability to generate bacteriocins, organic acids, and hydrogen peroxide as inhibitory compounds [44]. Lee et al. [45] described the combined supplement of salt with fermentation by a starter culture (*Bacillus polymyxa*), decreasing histamine and other BA formation in fish. 

The combination of two or more preservation methods (hurdle technology) often shows a greater inhibitory effect against the targeted microorganisms than any single treatment [46]. The application of modified atmospheres with low a_w_ and the addition of nisin extended the shelf-life of chilled fillets of gilthead seabream stored at 0–15 °C [47]. In particular, the modified atmosphere packaging (MAP) technique is based on the use of the three principal gases (i.e., %CO_2_, %O_2_, and %N_2_) inside the package and provides optimal conditions for the effective retardation of both microbiological and chemical processes [48]. The use of MAP and additives containing quercetin reduced the risk of BA production in Pacific white shrimp at 4 °C [49], whereas the shelf-life of striped red mullet was extended by MAP with ozone treatment [50]. The application of MAP together with ultra-violet (UV) radiation caused the reduction of putrescine concentrations during storage at 4 °C for 22 days in fillets of rainbow trout [51]. Also, Yew et al. [52] demonstrated strong reductions in histamine, cadaverine, and putrescine contents in Indian mackerel packaged with MAP (100% CO_2_) after 12 days of storage at 5 °C. Indeed, other authors [53] reported increases of some BAs when the association of vacuum package and UV treatment was applied in fillets of tambacu during storage at 4 °C for six days. Two different doses of gamma radiation were investigated in samples of sea bream stored in ice, obtaining different results according to BAs. When increases in agmatine, tryptamine, and spermine were observed, cadaverine and putrescine levels decreased [54]. 

High pressure processing (HPP) is another technique able to inactivate microorganisms and autolytic enzymes at low temperature, thus extending the shelf-life of fish products [55]. The effect of HPP on BA formation was studied by Doeun et al. [56] in half-dried fish at different temperatures for 28 days. The authors observed a decrease of cadaverine and spermidine, while tyramine and spermine increased in concentration. 

Many bioactive compounds deriving from plants were investigated for their use against pathogens and spoilage microorganisms [57]. Essential oils (EOs) are produced by different part of plants and consist of complex mixtures of hundreds of individual aromatic volatile oily compounds [58], even if only 300 are used in the food industry [59]. They are distinguished into several groups (i.e., terpenes, terpenoids, aromatics, and other compounds) according to their chemical structure [60]. According to the literature, EOs from oregano, rosemary, thyme, laurel, sage, cinnamon, clove, and basil are the most described antimicrobial and antioxidant agents in fish and fishery products. They can be applied to inhibit bacterial growth or for their bactericidal actions at high concentrations [61]. With regard to their effect on BAs, Özogul et al. [62] reported that rosemary and sage tea extracts could reduce histamine, putrescine, and cadaverine content in fillets of sardine during storage at 3 °C, 100 times smaller than the control group. Similarly, Cai et al. [63] found lower histamine levels in fillets of red drum stored at 4 °C treated with cumin, clove, and spearmint as essential oils. The authors supposed that such treatment inhibited the growth of microorganisms with histidine decarboxylase activity. Vacuum-packed fillets of sardine were stored after the addition of ethanolic extracts from mint and artemisia at 3 °C for 21 days. The contents of histamine, tyramine, and cadaverine were lower in treated than in control samples, and extracts of mint were more efficient than artemisia [64]. Kuley et al. [65] evaluated the inhibitory effects of safflower and bitter lemon extracts on both fish spoilage and growth of pathogenic bacteria. Such effects varied depending on the bacterial strains and specific amines. A general decrease in BA accumulation was observed and histamine production by *P*. *phosphoreum* was considerably suppressed. The effects of a microemulsion containing 0.3% or 1% lemon EO on the quality of salted sardines during 150 days of ripening were reported by Alfonzo et al. [66]. The results showed a reduction in *Enterobacteriaceae*, staphylococci, and rod LAB counts and a lower accumulation of histamine in the treated sardines compared to the control. However, as some EOs can have a negative impact on the sensory characteristics of seafood, even at low doses, some authors suggested the use of edible coating films enriched with EOs as an alternative and interesting option in order to reduce the required doses [67,68]. A more recent approach is active food packaging, i.e., the incorporation of EOs into the food package with a controlled release in order to maintain the organoleptic properties and microbiological integrity of food [69,70].

Also, other natural compounds, such as tea polyphenols and sage extracts, are used for food preservation [71,72,73]. The application of chitosan is becoming more frequent in the seafood industry due to its antibacterial and antioxidant characteristics [74,75].

## 4. Preventive and Control Measures

Fish products are subjected to spoilage because the chemical composition of meat rather than the microbial load on the skin or gastrointestinal tract can affect shelf-life, therefore, they must be chilled as soon as possible after catching. The formation of BAs in seafood is mostly dependent on time/temperature conditions from harvest through consumption. The best practices to control the growth of microorganisms producing BAs, histamine included, are chilling and freezing, even if it was reported that some microorganisms can grow at low temperatures and produce decarboxylases [76]. 

Many preservation processes, such as freezing, drying, margination, or salting can control the development of spoilage bacteria in fish [44]. The heating treatment before canning can eliminate both histamine-producing bacteria and their enzymes, but as histamine is heat stable, it can be found in the final product because it was present before the technological process started. Moreover, histamine can be still produced after thermal treatment when temperature abuse or recontamination appear [12]. Therefore, a dual approach for the control of BA increase in seafood is based on the quality of raw material, as well as the implementation of specific conditions able to inhibit or eliminate microorganisms with potential BA formation activity [77]. 

The prevention of BA formation in raw fish is primarily based on the rapid cooling after catching and its subsequent storage at ice temperature, as well as correct handling and hygiene practices on-board vessels. Ice, slurry ice, or mechanically refrigerated sea water can be used to chill fish after harvesting. The amount of ice used, as well as the size and temperature of fish brought on board and the air temperature on the deck and in storage hold, can affect the rate at which the internal temperature of fish refrigerate. For instance, it is well known that large fish chill more slowly than small fish. Moreover, the practice of placing ice into the gut cavity of fish could allow faster refrigeration, but if it is inappropriate, it can cause contamination from bacteria from the visceral cavity to the flesh fish and consequently the process of histamine production is accelerated. According to FDA recommendations (revised guidance, year 2020), if the water temperature is above 28.3 °C, the caught fish must be put into ice or in refrigerated seawater at 4.4 °C as soon as possible; instead, if the water temperature is below 28.3 °C the fish must be chilled within a maximum of 9 h from death. This period corresponds to 12 h if the fish are gilled and gutted before chilling [23]. 

According to the EU legislation (Regulation EC No 853/2004, currently in force) fish and fishery products must be kept at temperature of the melting ice as soon as possible after catching and during steps such as production, transport, storage, and distribution. Also, the operations of heading and gutting must be carried out quickly, and then whole and gutted fresh fishery products must be stored at ice temperature, while frozen fishery products must be kept at a temperature of no more than −18 °C. 

All these requirements are necessary not only to ensure fish freshness and quality, but also to avoid potential histamine formation in fish families with a high histidine levels in muscle tissue. Indeed, fishermen and all food business operators involved in the fish food chain must ensure that the regulatory limits of histamine are not exceeded. 

With regard to processed fishery products, it is known that some histamine-forming microorganisms are halotolerant or halophilic and can produce histamine at low pH levels. Moreover, technological processes such as salting, drying, fermenting, smoking, and pickling can contribute to histamine formation. For fermented seafood, the addition of negative amine-producing starter cultures could prevent BA formation during processing and storage [21]. 

Many regulatory organizations around the world adopted maximum limits for histamine in fish and fishery products. The EU, the Codex Alimentarius, Australia, and New Zealand established histamine values above 200 mg/kg as a maximum limit in raw fish, whereas the FDA considered 50 mg/kg for United States and Canada [78]. The last value was derived from the different distribution of amines in large fish, i.e., if in one section of a fish the histamine concentration is 50 ppm, in other parts of the same fish 500 ppm can be found [23]. Instead, in the Russian Federation the histamine maximum content is 100 mg/kg for salmon, herring, tuna, and mackerel [79], according to “The Sanitary and Epidemiological Rules and Regulations, SanPin 2.3.2.1078-2001”. 

Sampling and analysis also constitute important components of the control strategy. In Table 6, the sampling plan for histamine determination according to the Commission Regulation (EC) No 2073/2005 and FDA Guidance is well described. The main difference regards the number of samples to be analyzed. The collection and analysis of 18 samples described in the FDA scheme can allow the detection of nonconforming lots with a higher probability than the EU sampling plan [80]. Instead, the last scheme consists of nine samples, except for fish collected at the retail level, where only a single sample can be analyzed; if the result is higher than the maximum limit (200 mg/kg), the whole batch is considered unsafe. Also, for fish sauce produced by fermentation of fishery products, one sample can be considered representative, because histamine is uniformly distributed in these products.

The Commission Regulation (EC) No 2073/2005 also establishes the analytical reference method (EN ISO 19343:2017) for histamine detection in fish by high-performance liquid chromatography (HPLC). 

## 5. Trend and Challenges of Detection Technologies 

The detection of BA in fish is generally difficult, requiring long time of analysis and expert technicians. Moreover, a phase of preconcentration is often necessary for complex matrices and the separation of histamine from interference compounds, such as histidine or carnosine, needs careful and long pretreatment of the sample with precolumn or postcolumn derivatization, which is time-consuming and prolongs the entire analytical process [81]. Some other hurdles are described for BA determination, such as strong polar characteristics, the simultaneous occurrence of several BAs, and variable concentration ranges [82].

Many simple and rapid techniques are described in the literature for monitoring histamine levels in fish and fishery products, such as biosensors, immune-enzymatic assays (ELISA), and colorimetric methods. Alonso-Lomillo et al. [83] reported the use of amine oxidase-based electrodes to catalyze the oxidative deamination of BAs producing other compounds, such as ammonia and hydrogen peroxide. The commercial ELISA kits are functional as screening methods due to their quickness and simplicity, even if they give only semiquantitative results [84]. Also, colorimetric methods can be applied for routine analysis [85], as well as capillary electrophoresis, which is less sensitive than other methods but is rapid and cheap, allowing the analysis of many samples in a short time [82]. 

In the fishery industry and on an own-check basis, such simple detection technologies used as screening tests must be reinforced by confirmatory methods if positive results are achieved. Among them, thin-layer chromatography [86], gas chromatography [87], and electrochemical assays [88] are known to be sensitive and specific but require trained technicians and sophisticated, expensive instruments [89]. In particular, thin-layer chromatography is a technique that allows the fast determination of small quantities of compounds, as well as the simultaneous analysis of many samples. With regard to gas chromatography, some analytical problems were reported and it is better to use it in combination with derivative technologies. Finally, the electrochemical detectors are rapid and easy tests with high sensitivity [90]. 

In recent years, new technologies based on nanomaterials, such as carbon nanotubes [91], graphene [92], and metal nanoparticles [93,94], significantly improved the speed and cost of analysis, low sample volume requirement and field deployability. The use of gold nanoparticles for the detection of two important volatile biogenic markers, i.e., dimethyl sulfide and histamine, was described by Chow et al. [95], showing excellent selectivity in the presence of other volatiles commonly produced during fish spoilage and a low limit of detection of 0.5 and 0.035 μg/mL, respectively. A dual detection approach based on colorimetric sensor and laser desorption–ionization mass spectrometry was also reported by Siripongpreda et al. [96] for screening and quantitative determination of BAs. 

## 6. Conclusions

In fish and fishery products, BAs can be considered as indicators of both quality and safety, and their formation depends on the harvest method, the handling and other operations onboard vessels, post catching contamination, inadequate chilling, or temperature abuse. In fish exposed to high temperatures even for a short time, large populations of microorganisms can grow and produce decarboxylases. Even if during the subsequent refrigeration bacterial growth is reduced, residual enzyme activity can continue and, therefore, BA levels increase. The application of appropriate preventive strategies and control procedures represent the most efficient tool for both consumers and the fish industry.

## Figures and Tables

**Table 1:** Classification of biogenic amines based on chemical structure.

Precursors	Biogenic Amines		
	Aliphatic	Aromatic	Heterocyclic
Arginine	Agmatine		
Lysine	Cadaverine		
Ornithine	Putrescine		
Phenylalanine		Phenylethylamine	
Tyrosine		Tyramine	
Histidine			Histamine
Tryptophan			Tryptamine

**Table 2:** Microorganisms producing biogenic amines in seafood.

Biogenic Amine	Microorganisms	References
Histamine	*Morganella morganii*, *Morganella psychrotolerans*, *Hafnia alvei*, *Photobacterium phosphoreum, Photobacterium psychrotolerans, Klebsiella pneumoniae*, *Clostridium* spp., *Pseudomonas fluorescens*, *Pseudomonas putida*, *Pseudomonas cepaciae*, *Aeromonas* spp., *Aeromonas hydrophila*, *Acinetobacter lowffi, Plesiomonas shigelloides,* *Proteus vulgaris*, *Proteus mirabilis*, *Serratia fonticola*, *Serratia liquefaciens*, *Enterobacter cloacae*, *Enterobacter aerogenes*, *Klebsiella oxytoca, Citrobacter freundii*, *Raoultella planticola, Staphylococcus xylosus, Staphylococcus epidermidis, Bacillus* spp., *Vibrio alginolyticus*, *Vibrio* spp., *Escherichia* spp.	Hungerforf, 2010 [5] Biji et al., 2016 [2] Doeun et al., 2017 [6] Barbieri et al., 2019 [7] Xu et al., 2020 [8]
Tiramine	Lactic acid bacteria (including *lactobacilli*, *lactococci*, *enterococci* and *carnobacteria*)	Marcobal et al., 2012 [9]
Putrescine	*Enterobacter* spp., *Hafnia alvei*, *Pantoea agglomerans*, *Serratia liquefaciens*, *Photobacterium phosphoreum*, *Aeromonas* spp., *Lactobacillus curvatus*, *Lactobacillus sakei*, *Carnobacterium divergens*	Wunderlichová et al., 2014 [10]
Cadaverine	Pseudomonads, *Enterobacteriaceae*	Paleologos et al., 2004 [11] Kuley et al., 2017 [4]

**Table 3:** Symptoms of scombroid poisoning.

Apparatus	Symptoms
Integumentary	Face, neck, and upper arm flushing, itchy rash, hives, localized swelling, redness, urticaria, pruritus
Cardiovascular	Hypotension with distributive shock, cardiac arrhythmias, myocardial disfunction, acute pulmonary edema, oral numbness, tingling
Gastrointestinal	Abdominal pain, stomach cramps, nausea, vomiting, diarrhea
Neurological	Throbbing headache, migraines, loss of sight, dizziness, faintness, anxiety, tremor
Respiratory	Asthma attacks, respiratory distress, rhinitis, bronchoconstriction, dyspnea
Other	Metallic or peppery taste, oral numbness, difficulties in swallowing and thirst, feeling of warmth around the mouth

**Table 4:** Number of histamine poisoning outbreaks reported in the European Union during the years 2010–2018 [27,28,29,30,31,32,33,34,35].

Year	Outbreaks (N)			Cases (N)	
	Strong-Evidence	Weak-Evidence	Total	Human Cases	Hospitalized
2018	24	56	80	488	115
2017	56	61	117	572	51
2016 *	28	78	106	489	74
2015	23	57	80	437	43
2014	35	- **	35	164	15
2013	42	-	42	231	30
2012	34	-	34	241	14
2011	58	-	58	259	31
2010	33	-	33	185	12

Legend: * Data reported as other causative agents that include chemical agents, histamine, lectin, marine biotoxins, mushroom toxins, and scombrotoxin; ** data of weak-evidence outbreaks not reported.

**Table 5:** Histamine concentrations reported in “fish and fish products” by the Rapid Alert System for Food and Feed from 1 January 2015 to 31 August 2020.

Notification	Histamine Concentrations (mg/kg)					
	≤200	>200 and ≤500	>500 and ≤1000	>1000 and ≤2000	>2000	Total
IA *	31 (20.6%)	52 (34.4%)	23 (15.2%)	29 (19.2%)	16 (10.6%)	151 (100%)
Alert	15 (12.9%)	35 (30.2%)	17 (14.7%)	26 (22.4%)	23 (19.8%)	116 (100%)
BR **	8 (22.2%)	18 (50%)	9 (25%)	-	1 (2.8%)	36 (100%)

Legend: * information for attention; ** border rejection.

**Table 6:** Sampling plan for histamine determination according to official control rules.

Country	Limit	Reference
EU	Fishery products from fish species associated with a high amount of histidine n = 9, c = 2, m = 100 mg/kg, M = 200 mg/kg	Commission Regulation (EC) No 2073/2005 and further amendments
	Fishery products undergoing enzyme maturation treatment in brine, manufactured from fish species associated with a high amount of histidine n = 9 *, c = 2, m = 200 mg/kg, M = 400 mg/kg	
	Fish sauce produced by fermentation of fishery products n = 1, m = 400 mg/kg	Commission Regulation (EU) No 1019/2013
US FDA	n = 18, c = 1, m = 50 mg/kg, M = 500 mg/kg n = 18; c = 0, m = 500 mg/kg	FAO/WHO, 2012

Legend: * Single samples may be taken at retail level. If one of nine samples analyzed is found to be above M, the whole batch shall be deemed unsafe (Commission Regulation EU No 1019/2013).
